# Re-escalation of Treatment in Older Adults with Rheumatoid Arthritis After Anti-TNF Therapy De-escalation

**DOI:** 10.1093/geroni/igaf122.2417

**Published:** 2025-12-31

**Authors:** Jiha Lee, Jonathan Martindale, Una Makris, Julie Bynum

**Affiliations:** University of Michigan, Ann Arbor, Michigan, United States; University of Michigan, Ann Arbor, Michigan, United States; UT Southwestern Medical Center, Dallas, Texas, United States; University of Michigan, Ann Arbor, Michigan, United States

## Abstract

Older adults with rheumatoid arthritis (RA) treated with biologic disease-modifying antirheumatic drugs (bDMARDs), including anti-TNFs, are at an increased risk of serious infections and other adverse effects. Guidelines recommend de-escalating DMARDs for patients with low disease activity or remission to optimize the benefit-harm ratio of treatment. In a prior study, we observed that 20% of older adults with RA de-escalated anti-TNFs. This follow-up study examines the prevalence and factors associated with the re-escalation of treatment after anti-TNF de-escalation. We used 20% Medicare data from 2009-2017 to identify RA patients ≥66 years of age on anti-TNF therapy (Adalimumab, Etanercept, Certolizumab, or Golimumab), with de-escalation by cessation (> 90-day gap) or by taper (>50% effective dose reduction), and at least one rheumatologist visit pre- post-de-escalation (i.e. index-date). Re-escalation was defined as restarting the same anti-TNF or another bDMARD or increasing the effective dose by > 50% after the index date. Information on baseline patient characteristics, concomitant use of conventional synthetic DMARDs (csDMARDs), and changes in average glucocorticoids (GC) dose were collected. We identified 814 eligible Medicare beneficiaries, with an average age of 75.2 (SD 5.8), 84.5% female, 75.8% non-Hispanic white, and 43.4% with low-income subsidies (LIS). Of them, 61.1% re-escalated treatment with a median time of 160 (IQR 112-290) days since the index-date. In Cox analyses, re-escalation was more likely among blacks, Hispanics/others, and those with lower comorbidity burdens. Overall, more than half of older RA patients re-escalate treatment within a year of anti-TNF de-escalation highlights the importance of bDMARDs for disease control.

